# Adsorption Ability of Graphene Aerogel and Reduced Graphene Aerogel toward 2,4-D Herbicide and Salicylic Acid

**DOI:** 10.3390/gels9090680

**Published:** 2023-08-23

**Authors:** Alexandra Yu. Kurmysheva, Oleg Yanushevich, Natella Krikheli, Olga Kramar, Marina D. Vedenyapina, Pavel Podrabinnik, Nestor Washington Solís Pinargote, Anton Smirnov, Ekaterina Kuznetsova, Vladislav V. Malyavin, Pavel Peretyagin, Sergey N. Grigoriev

**Affiliations:** 1 Laboratory of Electric Current Assisted Sintering Technologies, Moscow State University of Technology “STANKIN”, Vadkovsky per. 1, 127055 Moscow, Russia; p.podrabinnik@stankin.ru (P.P.); nw.solis@stankin.ru (N.W.S.P.); a.smirnov@stankin.ru (A.S.); e.kuznetsova@stankin.ru (E.K.); p.peretyagin@stankin.ru (P.P.); s.grigoriev@stankin.ru (S.N.G.); 2 Scientific Department, A.I. Yevdokimov Moscow State University of Medicine and Dentistry, Delegatskaya St., 20, p. 1, 127473 Moscow, Russia; olegyanushevich@mail.ru (O.Y.); nataly0088@mail.ru (N.K.); dr.ovkramar@gmail.com (O.K.); 3 N. D. Zelinsky Institute of Organic Chemistry, Russian Academy of Sciences, Leninsky Prospect 47, 119991 Moscow, Russia; mvedenyapina@yandex.ru; 4 Laboratory of Petroleum Chemistry and Petrochemical Synthesis, Topchiev Institute of Petrochemical Synthesis, Russian Academy of Sciences, Leninsky Prospect 29, 119991 Moscow, Russia; malyavin@ips.ac.ru

**Keywords:** graphene oxide aerogel, reduced graphene oxide aerogel, 2,4-D, salicylic acid, adsorption, water treatment

## Abstract

Within this work, new aerogels based on graphene oxide are proposed to adsorb salicylic acid (SA) and herbicide 2,4-Dichlorophenoxyacetic acid (2,4-D) from aqueous media. Graphene oxide aerogel (GOA) and reduced graphene oxide aerogel (rGOA) were obtained by freeze-drying processes and then studied by Raman spectroscopy, Fourier-transform infrared spectroscopy (FT-IR), and Brunauer–Emmett–Teller (BET) analysis. The influence of contact time and the concentration of the adsorbates were also assessed. It was found that equilibrium for high adsorption is reached in 150 min. In a single system, the pseudo-first-order, pseudo-second-order kinetic models, Intraparticle diffusion, and Elovich models were used to discuss the detail of the aerogel adsorbing pollutant. Moreover, the Langmuir, Freundlich, and Temkin adsorption models were applied to describe the equilibrium isotherms and calculate the isotherm constants.

## 1. Introduction

In recent years, the number of hazardous organic compounds entering natural water systems has increased dramatically with urban and industrial development. Among the organic pollutants, biologically active organic compounds (BAOCs) pose a significant threat to the normal functioning of natural aquatic ecosystems. For example, 2,4-Dichlorophenoxyacetic acid (2,4-D) is a popular agricultural herbicide and also is a common environmental pollutant [1]. Due to poor biodegradability, low soil absorption coefficient, and high water solubility, 2,4-D, after application, leaches into the soil and then into the surface and groundwater [2]. In addition, 2,4-D affects not only weed plants but also may curb growth rates, cause problems with the reproductive system, and provoke deviations in behavior or death of organisms [1]. Furthermore, 2,4-D is toxic to the human central nervous system, kidneys, and liver [3].

Another group of BAOCs includes medicines and, particularly, salicylic acid (SA), which is one of the most widespread compounds for analgesic and antipyretic agents [4]. After use, SA is excreted from the body as the parent drug and its metabolites and eventually ends up in sewers and wastewater treatment plants [5]. Because SA is not completely removed by conventional wastewater treatment processes; it is discharged into the receiving environment along with effluent and sludge [6]. SA is a highly toxic BAOC and causes serious environmental pollution [4].

To extract SA and 2,4-D from aquatic environments, different materials such as polymers [7,8], biochar [9,10], metal–organic frameworks [11,12], and activated carbon [13,14,15,16] were used. Among the various adsorbing materials, graphene oxide (GO) is a promising compound, as it is reported that oxygen-containing functional groups on the surface of GO can act as active adsorption sites for various pollutants [17]. However, 2-dimensional GO has poor structural stability in aquatic environments making it difficult to retrieve and reuse [18]. Three-dimensional structures (sponges, aerogels) can preserve GO features while allowing multiple reuses while in service [19]. Advanced GO-based aerogels (GOAs) are applied in many fields, including medicine [20,21,22].

Both reduced and non-reduced GOAs are well-studied and known as powerful adsorbents for extracting different oils [23,24], organic colorants [25,26,27,28], and metals [29,30,31,32] from aquatic environments. Therefore, it is of interest to study the adsorption capacity of GOA for SA and 2,4-D.

The work aims to study the adsorption capacity of GOAs in reduced and non-reduced forms for SA and 2,4-D. For this purpose, samples of graphene oxide aerogel and reduced graphene oxide aerogel (rGOA) were produced. The specific surface area was measured, and Raman and FT-IR studies were carried out. Adsorption rates were calculated by pseudo-first and pseudo-second-order kinetic models, and adsorption isotherms were described by Langmuir, Freundlich, and Temkin models.

## 2. Results and Discussion

### 2.1. Characterization of Adsorbents

**Figure 1:**
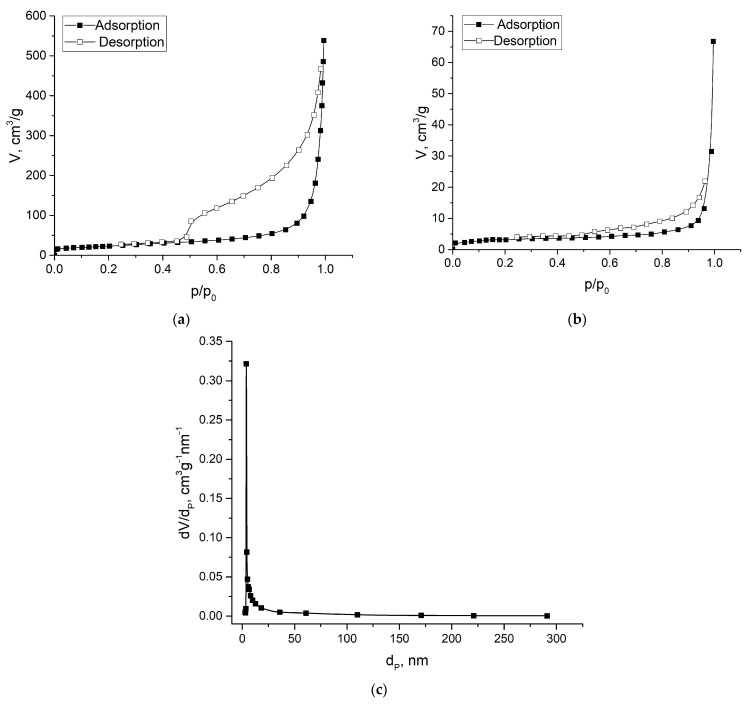
The nitrogen adsorption-desorption isotherms of GOA (**a**), rGOA (**b**), and pore size distribution of GOA (**c**).

Figure 1 illustrates N_2_ adsorption–desorption isotherms that were conducted on graphene oxide aerogels to determine their specific surface areas and pore sizes. The adsorption/desorption behavior of nitrogen on GOA and rGOA was typical for type IV with an isothermal H3 hysteresis loop indicating the presence of a mesoporous structure in the samples. The specific surface area measured by the BET method (S_BET_) for GOA and rGOA was 80.1 and 11.9 m^2^/g, respectively, while the total pore volume was 0.67 and 0.06 cm^3^/g. For the GOA sample, following the pore size distribution diagram (Figure 1c), pore sizes ranged from 5 nm to 16 nm, proving that GOA had a mesoporous structure. The average pore size of the GOA sample was 11.6 nm. The average pore diameter of the rGOA sample was impossible to calculate due to the low total pore volume.

The specific surface area of the rGOA was much smaller than that of GOA, which may be due to the adhesion of graphene during the reduction of GO with hydrazine [33] and the agglomeration of the sample during the BET study [34].

**Figure 2:**
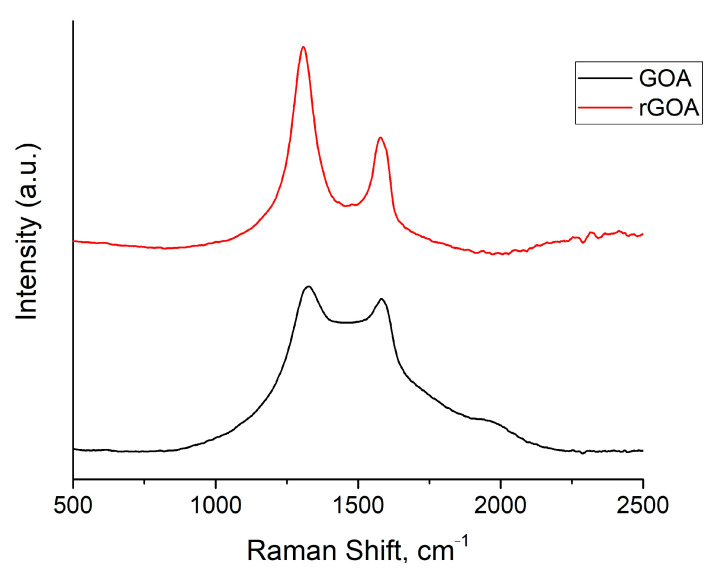
Raman spectra of GOA and rGOA.

Raman spectroscopy was used to examine structural changes in the GOA and rGOA samples after reduction (Figure 2). The obtained results show that the GOA sample has two characteristic peaks at 1350 cm^−1^ and 1582 cm^−1^, indicating D and G bands, respectively. Normally, the D band is disordered owing to structural defects, edge effects, and dangling sp^2^ carbon bonds, while the G band is due to the in-plane bond stretching of the sp^2^ carbons [35]. For the GOA sample, the G band is usually found at 1582 cm^−1^. After reduction, it was shifted to a lower wavenumber of 1580 cm^−1^, suggesting overlapping or sticking of several layers of graphene on top of each other after reduction with hydrazine hydrate [36,37,38]. This opinion is also supported by the results of specific surface area measurements performed by BET. Importantly, the intensity ratio of D and G bands for non-reduced forms was 1.08 against 1.86 for the rGOA, which is 1.7 times less. Thus, it can be concluded that newly formed sp^2^-hybrid domains in the reduced GOA are smaller in size than in the non-reduced one but more widespread [39].

**Figure 3:**
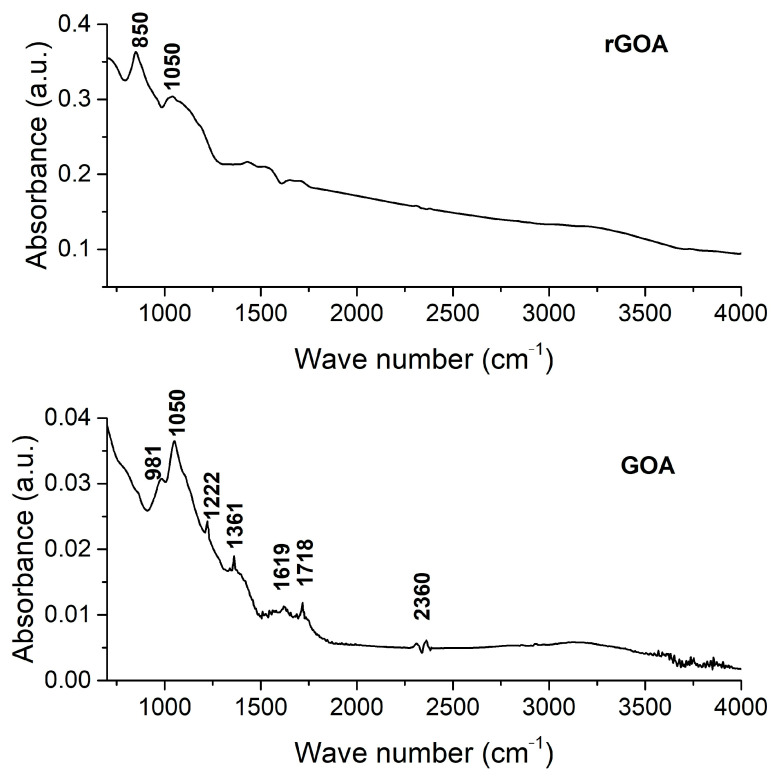
FT-IR spectra of GOA and rGOA.

The obtained compounds were also studied by FT-IR spectroscopy by identifying the molecular groups’ structures before and after the reduction process. The FT-IR spectrum of the aerogels is shown in Figure 3.

Since the samples were thoroughly dried, no significant signals belonging to water molecules encapsulated within the structure and stretching vibration of hydroxyl groups, theoretically located around 3500–3000 cm^−1^, were almost completely removed [40]. A weak peak observed on the GOA spectrum at 2360 cm^−1^ can be attributed to stretching vibrations of the C–H bond [41]. Peaks at 1718 cm^−1^ of the GOA sample correspond to the stretching vibrations of carbonyl groups [42], while peaks at 1619 cm^−1^ could be associated with the C=O stretching vibrations from carbonyl or carboxyl groups [43]. The peaks at 1361 cm^−1^ and 1222 cm^−1^ correspond to the stretching vibrations of the C=O and C–O bond, respectively [44]. The peak at 1050 cm^−1^ can be attributed to the C–O–C stretching vibration of the alkoxy group [45]. According to [46], a peak at 981 cm^−1^ is assigned to the ring out-of-plane deformation. As seen in Figure 3, the peak intensities of the rGOA oxygen-containing functional groups decreased compared to GOA, and some are gone completely. Thus, the reduction of GO by hydrazine hydrate was successful. Along with that, a new peak at 850 cm^−1^, identified as the C–O–C epoxy group, was found in the rGOA spectrum. [47]. As reported in [48], the presence of surface functional groups is critical for adsorbents removing 2,4-D and SA from aqueous solutions.

**Figure 4:**
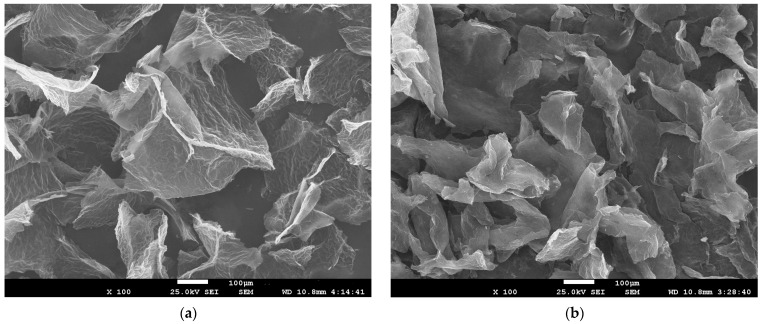
SEM images of GOA (**a**) and rGOA (**b**).

The morphology and the structure of the GOA and rGOA samples studied by scanning electron microscopy (SEM) are observed in Figure 4. Both samples demonstrate a wrinkled sheet structure [32]. However, compared to non-reduced aerogel, rGOA is characterized by smaller and more compacted sheets less distanced from each other. It suggests that such changes in morphology are attributed to the weakening of electrostatic repulsion between graphene nanosheets due to the removal of oxygen-containing groups during the reduction process [39].

### 2.2. Adsorption Kinetics

Defining the adsorption kinetics helps to understand the mass exchange mechanism during adsorption and specify the rate-limiting stage [24]. Adsorption kinetics were studied using the pseudo-first-order (PFO) [49], the pseudo-second-order (PSO) [50], the Elovich model [51], and the intraparticle diffusion model (IPD) [52]. The model parameters and correlation coefficients (R^2^) were obtained by nonlinear regression using Origin Pro 2016 software.

The pseudo-first-order model suggests that the rate of change in the absorption of solute materials over time is directly proportional to the difference in saturation concentration and amount of solid material absorbed over time. This assumption is usually applicable at the beginning of the process. Normally, adsorption kinetics follow this model if the adsorption process proceeds through diffusion through the phase boundary. The pseudo-first-order model can be described by the following equation:

(1) $$q_{t}=q_{e}\cdot \left(1-exp^{-k_{1}\cdot t}\right),$$

where $k_{1}$ (min^−1^) is the rate constant, and $q_{e}$ and $q_{t}$ (mg/g) are the number of adsorbed ions of 2,4-D or SA at equilibrium and at time t, respectively.

On the other hand, the pseudo-second-order model assumes that chemisorption is a rate-limiting stage of the adsorption process and predicts its behavior over the entire adsorption range. In this case, the adsorption rate depends on the adsorption capacity of the sorbent and not on the concentration of the adsorbate [50]. One of the main advantages of this model compared to the PFO model is that the PSO model can be used to calculate the equilibrium adsorption capacity at any time. The pseudo-second-order model equation can be represented as follows:

(2) $$q_{t}=\frac{q_{e}^{2}\cdot k_{2}\cdot t}{1+q_{e}^{2}\cdot k_{2}\cdot t},$$

where $k_{2}$ (g/(mg∙min)) is the rate constant in the PSO model.

The Elovich model is based on the hypothesis that the adsorption rate decreases exponentially as the amount of adsorbed solute material grows. It is expressed in the following way [51]:

(3) $$q_{t}=\frac{1}{\beta }\ln\left(\alpha \beta t+1\right),$$

where *α* (mg/g min)–initial adsorption rate; *β* (g/mg)–desorption constant during each experiment.

**Figure 5:**
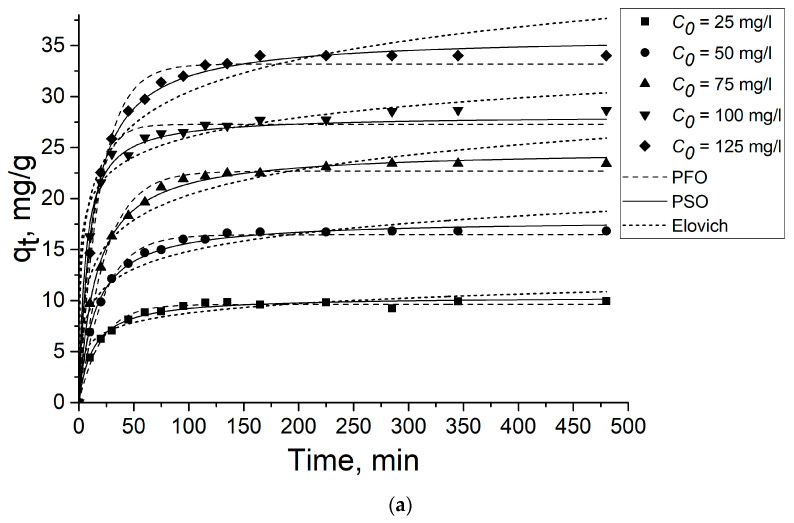

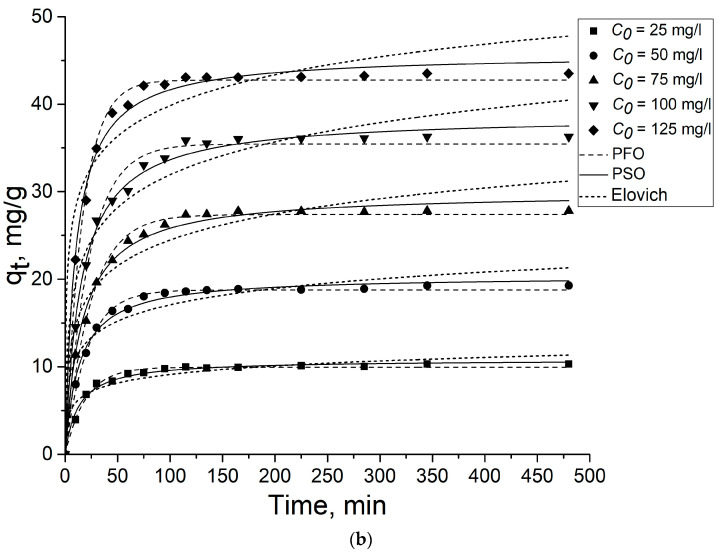
Effect of contact time on 2,4-D (**a**) and SA (**b**) adsorption on GOA. Dots are experimentally obtained data for various *C*_0_; lines are the results of model calculations.

**Figure 6:**
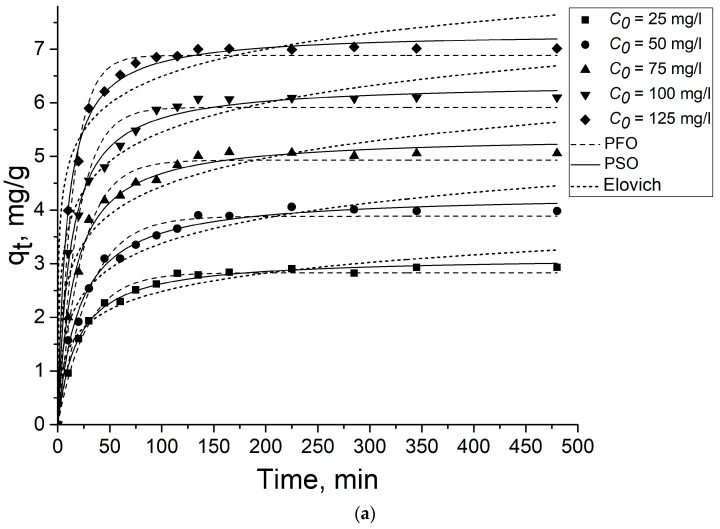

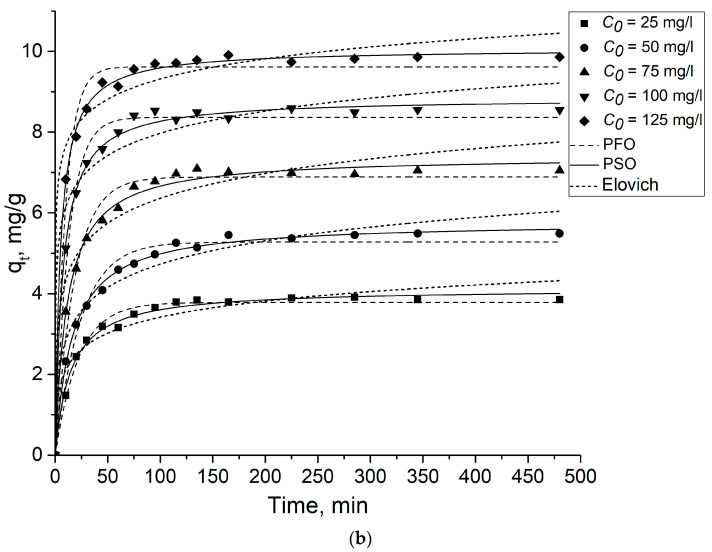
Effect of contact time on 2,4-D (**a**) and SA (**b**) adsorption on rGOA. Dots are experimentally obtained data for various *C*_0_; lines are the results of model calculations.

Figure 5 and Figure 6 present the experimental results on the 2,4-D and SA adsorption on the aerogel samples described by three models: PFO, PSO, and Elovich model. As seen from the figures, the adsorption equilibrium is achieved in less than 150 min. The parameters calculated according to the models are shown in Table 1 and Table 2. The PSO model gives the best fit with higher regression coefficients (R^2^) (>98%) for both 2,4-D and SA for both aerogel samples, signalizing that chemisorption is the adsorption rate-limiting step [53].

The kinetic process is directed by various mechanisms and stages of the adsorption phenomena. Usually, there are four main rate-limiting stages [54]: (1) volumetric diffusion, in which the adsorbate is transferred from the volume of the solution to the liquid film surrounding the solid adsorbent; (2) mass transfer of a solute from the boundary film to the surface of the adsorbent, also called external diffusion; (3) intraparticulate diffusion of solute material in the pores of the adsorbent. The porosity of the adsorbent is a key factor for the stage; (4) interaction with surface areas through chemisorption. Both the first and second stages can be attributed to film diffusion. The third stage is the stage of resistance to the internal diffusion of particles. The IPD model serves to evaluate the influence of the third adsorption step on the rate of the entire adsorption process and is studied by applying the Weber and Morris model as shown in Equation (4):

(4) $$q_{t}=k_{pi}\cdot t^{0.5}+C$$

where $k_{pi}$ (mg/g min^0.5^) is the IPD rate constant; *t*^0.5^ is the square root of time; *C* (mg/g) is an intercept. According to Equation (5), if the kinetic process is dominated by the intraparticle diffusion, the plot of *q_t_* versus *t*^0.5^ should be a straight line. In addition, if the intercept of the plot (*C*) is equal to 0, then the intraparticle diffusion is the only rate-limiting step [55].

**Figure 7:**
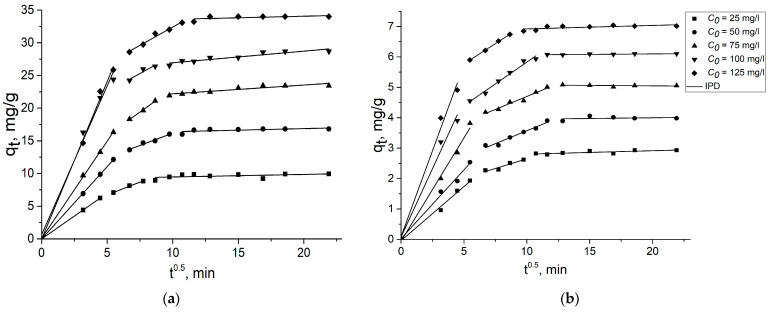

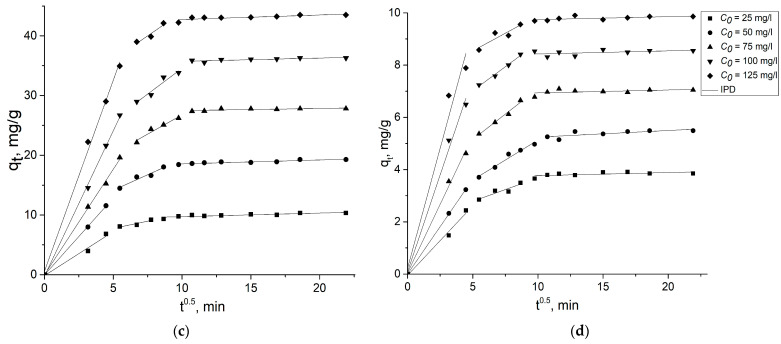
Intraparticle diffusion model of 2,4-D on GOA (**a**) and rGOA (**b**); SA on GOA (**c**) and rGOA (**d**).

As can be seen from Figure 7, the plots of *q_t_* versus *t*^0.5^ do not take a linear form during the entire adsorption time. This shows that the adsorption of 2,4-D and SA on GOA and rGOA does not follow the intraparticle diffusion model during the whole adsorption process. However, polylinearity is observed on the plots indicating the presence of two or more stages involved in the adsorption process. Moreover, each linear section of these graphs that deviates from the origin also shows that intraparticle diffusion is not the only rate-limiting stage.

According to [56], the *k_pi_* values can reflect the degree of contribution of initial sorption and intraparticle diffusion to the entire sorption process. At the first stage, in all cases, the best linearization with the corresponding first-rate constants IPD model *k_p_*_1_ (Table 3 and Table 4) was obtained, indicating the fastest process that can be associated with film diffusion. As a result, the hydrodynamic boundary layer was probably formed due to the diffusion of 2,4-D and SA molecules from the solution onto the outer surfaces of GOA and rGOA. In contrast, the second and last stages showed a decrease in the second- and third-rate constant of the IPD model (*k_p_*_2_ and *k_p_*_3_) with corresponding lower values of R^2^. The decrease in the order of the adsorption rate (*k_pi_*) in the sequence *k_p_*_1_ > *k_p_*_2_ > *k_p_*_3_ may be due to the resistance to a mass transfer occurring in the second phase of adsorption [56]. The second stage denotes a process in which the movement of adsorbate molecules can migrate from the outer surfaces of the adsorbent to the inner regions of the pores. The rate-limiting stage may be determined by a non-zero intersection calculated from the intersection (*C*) on the second area of the graph. In related studies, the calculated intersections (*C*) on the graphs were positive (>0), suggesting that the process showed slow diffusion within the particles, mainly due to the small concentration of adsorbate molecules remaining in the aqueous medium and the saturation of the remaining pores with molecules 2,4-D and SA [55].

### 2.3. Adsorption Isotherms

Three commonly used isotherm models, Langmuir [57], Freundlich [58], and Temkin [59] were adopted to depict the solid-liquid adsorption system.

The nonlinear form of the Langmuir isotherm could be described as follows:

(5) $$q_{e}=q_{m}b_{l}c_{e}/\left(1+b_{L}c_{e}\right),$$

where $q_{m}$—maximum capacity of the monolayer (mg/g), and $b_{l}$—the adsorption coefficient (L/g).

Mathematically, Freundlich isotherm can be presented as in Equation (4):

(6) $$q_{e}=K_{F}c_{e}{}^{1/n},$$

where $K_{F}$—the coefficient of distribution or adsorption coefficient (L/g).

Temkin isotherm is shown in Equation (5):

(7) $$q_{e}=\left(RT/b_{T}\right)\ln\left(Ac_{e}\right),$$

where $b_{T}$—the adsorption coefficient (J/mol); *R*—the universal gas constant of 8.314 J/(mol∙K); *A*—the constant, L/g; *T*—the absolute temperature (K).

Three kinds of isotherms have their own assumptions [24]: the Langmuir model assumes that the active sites from homogeneous surface share equal relations with the adsorbate. The hypothesis of the Freundlich model describes heterogeneous adsorption surfaces with different affinities of adsorption sites. The Temkin model assumes that the adsorption heat of adsorbate decreases linearly with the increase of coverage.

**Figure 8:**
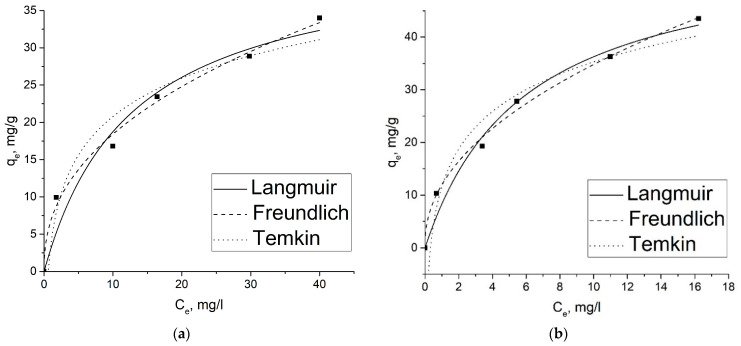
Plots of nonlinear isotherm models for the adsorption of 2,4-D (**a**) and SA (**b**) adsorption on GOA.

Figure 7a,b and Figure 8a,b show the results of applying the isotherm models to the experimental data of the adsorption of 2,4-D and SA ions on GOA and rGOA. Experimental and calculated parameters for each isotherm model are given in Table 5.

**Figure 9:**
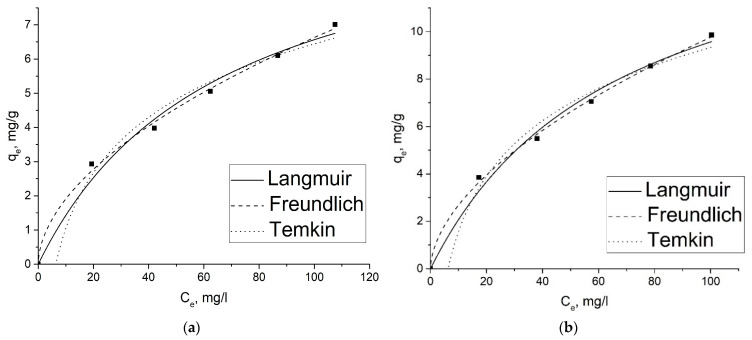
Plots of nonlinear isotherm models for the adsorption of 2,4-D (**a**) and SA (**b**) adsorption on rGOA.

As can be seen from Figure 8 and Figure 9, the capacity of the adsorbents with respect to SA is a bit higher than that of 2,4-D, which can be attributed to a smaller size of SA molecules.

It can be concluded from Table 5 that, according to the correlation coefficient (R^2^), the isotherm can be fitted with the Langmuir and Freundlich isotherms with high precision. However, the Freundlich fitting model appeared more representative than Langmuir one, comparing the R^2^ values. The adsorption process, according to the Langmuir model, must meet certain conditions, such as a homogeneous adsorbent surface, monolayer adsorption of the adsorbate on the adsorbent surface, and the absence of interaction between adsorbed particles [55]. However, some adsorption interactions do not always follow this rule. It may be a reason for the slight deviation of the 2,4-D and SA adsorption isotherms on aerogel samples from the Langmuir model in this work. The Freundlich isotherm is an empiric model, which is not limited to covering the surface of a sorbent by adsorbate molecules, but also describes multilayer adsorption and considers interactions between the adsorbed molecules. The Freundlich model constant *K_F_* reflects how favorable the adsorption is. The constant of the 1/*n* in the Freundlich model is related to the adsorption intensity, which varies with the heterogeneity of materials. Adsorption is favorable when the 1/*n* ratio is between 0 and 1 [55]. As seen from Table 5, the value of the 1/*n* ratio is between 0 and 1, indicating that the adsorption behavior of 2,4-D and SA on aerogels is favorable.

The maximum adsorption capacity is higher for GOA for both adsorbates than for rGOA, which may be due to the absence of oxygen-containing groups on the surface of rGOA. The maximum adsorption capacity and the rate of achieving the adsorption equilibrium of GOA in comparison with the results obtained in other works are presented in Table 6. On that background, the obtained results may be found relevant.

## 3. Conclusions

Within this work, both non-reduced and reduced with hydrazine hydrate GO-based aerogels were studied. Raman spectroscopy results confirmed the successful reduction process. The adsorption capacity of GOA for 2,4-D and SA was measured to be 42.63 mg/g and 57.61 mg/g, respectively, while rGOA showed only 10.92 mg/g and 16.01 mg/g. The difference in adsorption capacity may be attributed to the absence of oxygen-containing functional groups on the surface of rGOA, which are essential for the successful adsorption of the 2,4-D and SA molecules. The adsorption kinetics for both aerogel samples are consistent with the PSO model, indicating that the rate-limiting step may be chemical adsorption by sharing or exchanging electrons between the adsorbate and adsorbent.

Thus, the sorption ability of the herbicide 2,4-D and salicylic acid of aerogels based on graphene oxide was successfully confirmed in the work.

## 4. Materials and Methods

### 4.1. Materials and Reagents

All the chemicals in this study were of analytical grade and were used as received without any additional purification. Graphene oxide (GO) suspension (50 mg/mL) was provided by Graphenox (Moscow, Russia). The element composition of the GO suspension was provided by the manufacturer and included C–46%, O–49%, H–2.5%, and S < 1.5%. The hydrazine hydrate solution (100%), 2,4-Dichlorophenoxyacetic acid, and salicylic acid were purchased from Merck (Darmstadt, Germany). Hydrochloric acid HCl was provided by Himprom (Kemerovo, Russia).

### 4.2. Preparation of GOA and rGOA

**Figure 10:**
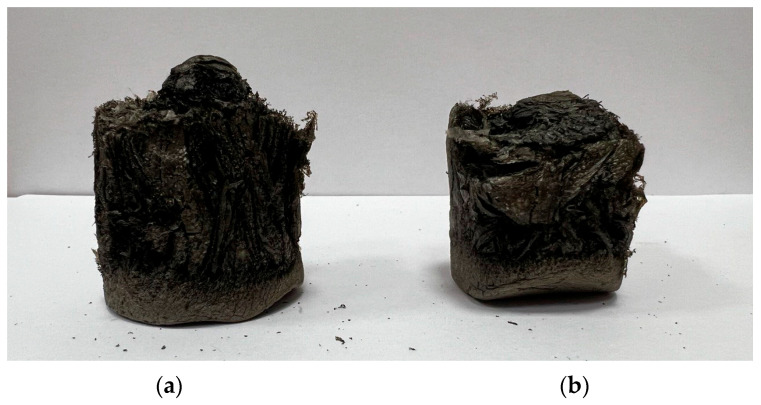
GOA (**a**) and rGOA (**b**) samples.

GO was dispersed into water by ultrasonication with a concentration of 3 mg/mL. The GO suspension was frozen at −70 °C and sublimated for 30 h by a freeze-dryer FreeZone 2.5 (Labconco, Kansas, MO, USA) to obtain the product. Further, the GOA samples were subjected to a chemical reduction in hydrazine vapor for 24 h to obtain rGOA. The obtained aerogels are presented in Figure 10.

### 4.3. Characterization of GOA and rGOA

Fourier-transform infrared spectroscopy (FTIR) spectra were obtained on a Vertex 70 infrared Fourier spectrometer (Bruker Optik GmbH, Ettlingen, Germany). The Raman spectra of graphene oxide and reduced graphene oxide aerogels were obtained on a DXRTM 2 Raman Micro-scope instrument (Thermo Fisher Scientific, Waltham, MA, USA) equipped with a 780 nm laser with a power of 15 mW. The laser beam was focused through the lens of an optical microscope with a magnification of 50× into a 0.8 μm spot on an area under investigation. The integration time for each spectrum was approximately 6 s. Scanning electron microscopy (SEM) images were obtained using SEM JEOL JSM-7001F (JEOL Ltd., Tokyo, Japan). The aerogel samples were preliminarily coated with a thin electric conductive gold film by an ion sputter coater Quorum Q150T Emscope sputter coater (Quorum Technologies Ltd., Newhaven, UK).

Nitrogen adsorption isotherms were recorded by using a BELSORP-miniX instrument (MicrotracBEL Japan, Inc. BELSORP-mini, Osaka, Japan). The preliminary preparation stage included thermal degasification of samples at 200 °C and 10 Pa pressure for 8 h. At the end of the preparation, the cuvette with the sample was cooled and weighed again to determine the weight loss of the sample after processing. After that, the cuvette was placed in the analytical port of the device, and the adsorption capacity was analyzed with the sequential supply of nitrogen until an equilibrium pressure in the system was reached at a temperature of 77 K in the relative pressure range p/p_0_ = 0–0.993. The total specific surface area was estimated according to the Brunauer–Emmett–Teller method (BET). The total pore volumes were estimated from a single point adsorption at p/p_0_ = 0.99.

### 4.4. Adsorption Experiments

The adsorption of SA и 2,4-D on graphene aerogel samples from aqueous solutions was carried out in a thermostated cell with a reflux condenser with nonstop mixing with a magnetic stirrer (150 rpm). The initial pH value of the solutions was set to 3 by adding the HCl solution dropwise. This pH value is optimal for better adsorption of 2,4-D and SA molecules in an acidic medium [1,2,3,4].

Adsorption kinetics were studied by tracking changes in the concentration of each adsorbate in time and the initial concentrations of 25–125 mg/L. The concentration of the SA and 2,4-D during the adsorption was determined by the collected specimens via UV spectroscopy from adsorption at 295 nm and 283 nm, respectively, on a Hitachi U-1900 instrument (Tokyo, Japan). The following Equation (8) was used to estimate the number of ions of SA and 2,4-D adsorbed per 1 g of graphene aerogel (mg/g):

(8) $$q_{e}=\frac{\left(C_{0}-C_{e}\right)\cdot V}{m},$$

where $C_{0}$ and $C_{e}$—initial and equilibrium concentration of each adsorbate ion, respectively; mg/L; V—volume of the solution, L; m—the mass of the adsorbent, g.

## Figures and Tables

**Table 1:** PFO and PSO kinetic models for the removal of 2,4-D and SA using GOA and rGOA.

*C*_0_, mg/L	Pseudo-First Order						Pseudo-Second Order					
	2,4-D			SA			2,4-D			SA		
	*k*_1_	*q_e_*_1_, mg/g	R^2^	*k*_1_	*q_e_*_1_, mg/g	R^2^	*k*_2_	*q_e_*_2_, mg/g	R^2^	*k*_2_	*q_e_*_2_, mg/g	R^2^
	GOA											
25	0.05	9.62	0.985	0.05	9.95	0.988	0.008	10.4	0.99	0.007	10.79	0.988
50	0.04	16.45	0.98	0.05	18.77	0.991	0.004	17.93	0.995	0.004	20.37	0.993
75	0.04	22.69	0.985	0.04	27.4	0.99	0.003	24.81	0.997	0.002	29.93	0.992
100	0.08	27.27	0.979	0.04	35.45	0.986	0.006	28.13	0.934	0.002	38.68	0.994
125	0.05	33.19	0.988	0.06	42.77	0.992	0.002	35.93	0.996	0.002	45.73	0.993
	**rGOA**											
25	0.04	2.83	0.986	0.05	3.78	0.984	0.016	3.13	0.993	0.017	4.13	0.991
50	0.03	3.88	0.974	0.04	5.28	0.974	0.011	4.29	0.99	0.014	5.78	0.996
75	0.04	4.93	0.984	0.05	6.89	0.978	0.012	5.39	0.991	0.012	7.41	0.994
100	0.05	5.92	0.963	0.08	8.37	0.985	0.013	6.38	0.992	0.016	8.84	0.996
125	0.07	6.88	0.986	0.1	9.62	0.981	0.017	7.32	0.996	0.02	10.06	0.998

**Table 2:** Elovich kinetic model for the removal of 2,4-D and SA using GOA and rGOA.

*C*_0_, mg/L	Elovich Equation					
	2,4-D			SA		
	*α*, mg/(g⋅min)	*β*, g/mg	R^2^	*α*, mg/(g⋅min)	*β*, g/mg	R^2^
**GOA**						
25	9.375	0.747	0.931	9.189	0.711	0.931
50	9.414	0.401	0.941	14.999	0.37	0.933
75	10.828	0.281	0.954	12.728	0.233	0.937
100	24.864	0.361	0.947	19.411	0.184	0.943
125	34.925	0.217	0.944	132.35	0.197	0.939
**rGOA**						
25	0.754	2.029	0.944	2.181	1.739	0.939
50	0.931	1.454	0.956	2.395	1.197	0.963
75	2.563	1.31	0.939	11.372	1.125	0.951
100	8.014	1.271	0.956	18.475	1.261	0.958
125	51.889	1.365	0.953	28.991	1.385	0.976

**Table 3:** Intraparticle diffusion constants of adsorption SA using GOA and rGOA.

*C*_0_ SA, mg/L	IPD								
	*k*_*p*1_, mg/g min^1/2^	*C*_1_, mg/g	R^2^	*k*_*p*2_, mg/g min^1/2^	*C*_2_, mg/g	R^2^	*k*_*p*3_, mg/g min^1/2^	*C*_3_, mg/g	R^2^
**GOA**									
25	1.475	−0.159	0.989	0.484	5.333	0.891	0.06	9.122	0.75
50	2.574	−0.036	0.999	1.036	8.967	0.939	0.065	17.909	0.867
75	3.531	−0.004	0.998	1.289	13.871	0.949	0.035	27.129	0.536
100	4.869	−0.246	0.999	1.728	17.284	0.933	0.055	35.181	0.683
125	6.399	0.569	0.996	1.586	28.101	0.919	0.076	41.986	0.653
**rGOA**									
25	0.531	−0.045	0.988	0.179	1.888	0.874	0.012	3.649	0.392
50	0.724	0.007	0.999	0.305	2.075	0.968	0.025	4.987	0.588
75	1.049	0.052	0.996	0.389	3.206	0.98	0.011	6.827	0.237
100	1.482	0.098	0.992	0.368	5.173	0.987	0.012	8.307	0.242
125	1.837	0.232	0.984	0.271	7.188	0.827	0.011	9.631	0.385

**Table 4:** Intraparticle diffusion constants of adsorption 2,4-D using GOA and rGOA.

*C*_0_ 2,4-D, mg/L	IPD								
	*k*_*p*1_, mg/g min^1/2^	*C*_1_, mg/g	R^2^	*k*_*p*2_, mg/g min^1/2^	*C*_2_, mg/g	R^2^	*k*_*p*3_, mg/g min^1/2^	*C*_3_, mg/g	R^2^
**GOA**									
25	1.396	−0.002	0.999	0.781	2.821	0.994	0.039	9.078	0.252
50	2.215	−0.027	0.999	0.75	8.668	0.974	0.047	15.913	0.432
75	2.973	0.077	0.999	1.436	8.627	0.996	0.134	20.847	0.853
100	4.553	0.634	0.989	1.113	16.949	0.911	0.177	25.161	0.867
125	4.824	−0.042	0.9995	1.117	21.223	0.974	0.048	33.09	0.331
**rGOA**									
25	0.35574	−0.043	0.99	0.126	1.388	0.911	0.011	2.688	0.635
50	0.45177	0.025	0.991	0.169	1.873	0.959	0.004	3.909	0.599
75	0.67995	−0.061	0.993	0.17	2.996	0.968	−0.003	5.102	0.119
100	0.8979	0.082	0.986	0.286	2.963	0.978	0.003	6.035	0.698
125	1.12817	0.095	0.988	0.267	4.432	0.998	0.012	6.804	0.466

**Table 5:** Isotherm parameters for 2,4-D and SA adsorption on GOA and rGOA.

Model Parameters		GOA		rGOA	
		2,4-D	SA	2,4-D	SA
Langmuir	*q_m_*, mg/g	42.63	57.61	10.92	16.01
	*b_l_*, L/g	0.08	0.17	0.015	0.015
	R^2^	0.982	0.98	0.987	0.99
Freundlich	*K_F_*, mg/g	6.86	11.9	0.55	0.73
	1/*n*	0.43	0.47	0.54	0.56
	R^2^	0.986	0.992	0.991	0.995
Temkin	*b_T_*, J/mol	333.43	241.21	1058.05	733.7
	*A*, L/g	1.64	3.1	0.16	0.16
	R^2^	0.963	0.966	0.983	0.984

**Table 6:** Comparison of capacity values for 2,4-D and SA ions sorbed by different adsorbents.

Adsorbent	Adsorbate	*q_e_*, mg/g	Equilibrium Time, min	Ref.
Magnetic Fe_3_O_4_@graphene nanocomposite	2,4-D	32.31	720	[60]
Polydopamine/polyacrylamide co-deposited magnetic sporopollenin		62.2	-	[61]
Algal magnetic activated carbon nanocomposite		60.61	-	[1]
GOA		42.63	150	This work
Chitosan-acrylamide surface molecularly imprinted hydrogel	SA	44.87	20	[4]
Biochar		36.39	720	[62]
Chitosan/xylan-coated magnetite nanoparticles		13.49	120	[63]
GOA		57.61	100	This work
